# Reflections on the Political Economy of European Wine Appellations

**DOI:** 10.1007/s40797-021-00145-4

**Published:** 2021-05-08

**Authors:** Julian M. Alston, Davide Gaeta

**Affiliations:** 1 Department of Agricultural and Resource Economics, University of California Davis, Davis, CA USA; 2 Robert Mondavi Institute Center for Wine Economics, University of California Davis, Davis, CA USA; 3 Department of Business Administration, University of Verona, Verona, Italy

**Keywords:** Collective reputation, Wine appellation, EU Common Market Organization, Geographic indications, Government failure, Q18, L51, L52, L66, N54

## Abstract

Today’s European wine policy is centered on a system of appellations, implemented as geographical indications (GIs), that entail significant technological regulations—restricting the varieties that may be grown, while imposing maximum yields per hectare and other rules regarding grape production and winemaking practice. This paper outlines the historical development of European wine policy under the CAP, and presents a more detailed analysis of the economic consequences of the rules and regulations under the appellation system. The introduction of these rules and regulations was probably beneficial initially, both for their didactive effect on wine producers and consumers and as a way of overcoming a significant “lemons” problem in the market. However, those same rules and regulations are much less valuable today, given (1) the potential for alternative sources of information to solve the lemons problem, and (2) evidence that the appellation system per se might not be effectively serving that purpose as well as it once did, while some of the regulations impose significant social costs. Yield restrictions, in particular, are economically inefficient as a way of enhancing and signaling quality (their ostensible purpose) and as a way of restricting total supply to support market prices and thus producer incomes (a significant motivation). The inherent weaknesses of the policy design are compounded by failures of governance. A less heavy-handed approach to policy would allow more scope for the market mechanism to match supply and demand for this signature product from European agriculture.

## Introduction

Around the world, wine markets are in a state of flux: producers are adjusting to shifting consumer demand for wine, global production is close to an all-time high, and we have once again entered a bust phase in the perennial boom-bust cycle—hard times for European wine producers, exacerbated by Brexit, President Trump’s tariffs, and now the COVID-19 pandemic. This evolving market context is one of several factors that has been putting pressure on Europe’s wine policy, which has sought for decades to manage the balance of supply and demand and quality of European wine, with only partial success.

Today’s European wine policy is centered on a system of appellations, implemented as geographical indications (GIs) for wine that entail significant technological regulations—restricting the varieties that may be grown, while imposing maximum yields per hectare and other rules regarding grape production and winemaking practice.^1^ This system was built over many decades on foundations laid in France in the early 1900s. The *Appellation d’Origine Contrôlée* (AOC) system was introduced in France in 1935 to establish standards for wine production systems—imposing regulations on both wine products and the processes used to produce them within defined locations—and thereby to create and enhance collective reputations based on regions (Haeck et al. 2019; Meloni et al. 2019). These regions and their typical wines were to be distinguished from one another by their unique combinations of terroir, varieties, standards, and production methods. The idea spread. In today’s global wine market as many as 1239 different wine appellations exist; 57 in Bordeaux alone (Livat et al. 2018; IOV 2019). Similar policies were introduced by other European wine-producing countries as they joined the European Community (EC), and the appellation rules were harmonized under the aegis of an expanding European Union (EU) throughout various reforms of the Common Agricultural Policy (CAP), most recently in 2013.

The creation of the AOC system in France (and its counterparts in other countries) may have yielded net social benefits by solving a “lemons” problem, as argued by Mérel et al. (2019), or through its didactic effects in educating poorly informed wine producers and consumers, or both. Over time, however, the cost–benefit balance has changed. Restrictions on producer choices over what to produce and how to produce it impose social costs that inevitably increase over time (Becker 1983). Exacerbating this general phenomenon, regulations on European wine production have been held largely immutable over decades during which technological and market possibilities for producers have changed immensely. And new demands for adaptive responses to climate change can only make matters worse. Meanwhile, improvements in marketing and communications technology, and the rise of alternative sources of information, have diminished the potential consumer and producer benefits from quality signals provided by wine appellations embodying information about production processes. And, with globalization, the world market has been substantially restructured, including a rise in New World wine market shares that may be partly attributed to the constraints imposed by the EU policies, and which changes the implications of those constraints. The policies have evolved, too, but perhaps too slowly.

The appellation system has operated in conjunction with a battery of supply management policies. Since the early 1980s, for many years the main concern for European wine policymakers was the problem of overproduction of undifferentiated table wines. However, as we explain below, this problem of excess capacity has now been transmitted to higher quality wines, produced within the system of GIs. These designations were introduced to provide a guarantee of excellence, to protect the consumer against risk of counterfeiting, and to serve as a symbol of the region of production for promoting the positive externalities from collective reputation. They may still do all these things, but the linkages between appellation rules and wine quality signals are becoming weaker, partly because the world has changed considerably since those rules were introduced. Some producers are finding it in their interest to opt out.

The objective of this paper is to explore the European system of appellations for wine, its management, the way in which it is regulated, its governance mechanisms, and the consequences for wine producers and consumers. In this context, we raise questions about the use of technological regulation as an economic policy tool. Economic arguments would favor instruments that are closely targeted to the economic purpose and that allow market transactions to reveal opportunity costs; ideas that have been neglected by EU wine policy.

Meloni et al. (2019) provide a taxonomy of wine regulations which they use to compare the EU and the other main producing countries. They note that “The EU is not only the largest global wine-producing region and the main importer and exporter of wine, but also the most regulated wine market” (Meloni et al., p. 622). As the authors highlight, Europe stands out from other producing countries for the size of its producer subsidies and the strength of its vineyard regulations seeking to increase the quality and restrict the quantity of wine produced. These vineyard regulations are the focus of the present paper, in particular the appellation rules and supply management policies. These policies are complex, involving diverse instruments devised and implemented by the EU, by Member States, and by sub-national organizations acting separately and together. Our analysis takes into account the realities of imperfect enforcement and economizing responses by producers and others.^2^

The paper proceeds as follows. First, in Sect. 2 we introduce the appellation rules that are the centerpiece of European wine policy and our main focus. Next, Sect. 3 presents a brief history of European policies as they evolved to address a persistent problem of surplus production of lower quality wine. Section 4 documents the resulting shifts in structure of the industry over the decades. Section 5 documents the consequences of various supply management policies, which has been a major emphasis of EU involvement, and a major public expense. Then we shift to the economics of appellations. Section 6 delves into GIs as collective brands. Section 7 analyzes firm-level economics of technological regulations as part of the appellation system, and Sect. 8 extends this analysis to the context of the market as a whole. Finally, Sect. 9 presents a synthesis of findings and concludes the article.

## Roots of European Wine Appellations

European wine law has evolved with the formation and expansion of the European Union, but its core elements have a century-long history. The main roots of core elements of today’s European wine policy can be traced to the “Great Wine Blight” in the late nineteenth century, caused by the introduction of the insect, phylloxera, which devastated vineyards around the world (Meloni and Swinnen 2013; Chevet et al. 2018). Eventually, the industry recovered by planting European *Vitis vinifera* stock grafted onto American phylloxera-resistant rootstock. But, meanwhile, responding to growing demand for wine that could not be satisfied from traditional sources, a large “French” wine industry had developed in Algeria; and fake wine or tampered wine was being sold (Meloni and Swinnen 2018). The upshot was several years of misery for French wine producers, culminating in a crisis in 1907, the climax of which was a demonstration in Montpellier by an estimated 600,000 to 800,000—one in every two residents of lower Languedoc—on June 9, 1907 (Munsie 2002; Chevet et al. 2018).

Some policy response was inevitable. Ultimately, building on precursor policies introduced between 1908 and 1911, France introduced its *Appellation d’Origine Contrôlée* (AOC) system in 1935, eventually to be supplanted by the EU in 2012 with its *Appellation d’Origin Protégée* system.^3^ Appellation rules closely define which grape varieties and winemaking practices are approved for classification in each of France’s several hundred geographically defined appellations, which can cover regions, villages or vineyards. They also specify restrictions on yields per hectare, vineyard area, and planting density, and thus total production for each appellation. This policy innovation spread to other European countries—in particular to those countries that joined what was eventually to become the EU.^4^ The ultimate outcome was a harmonized multinational system of GIs for wine, implemented in the context of substantial other government intervention in the market.

**Fig. 1:**
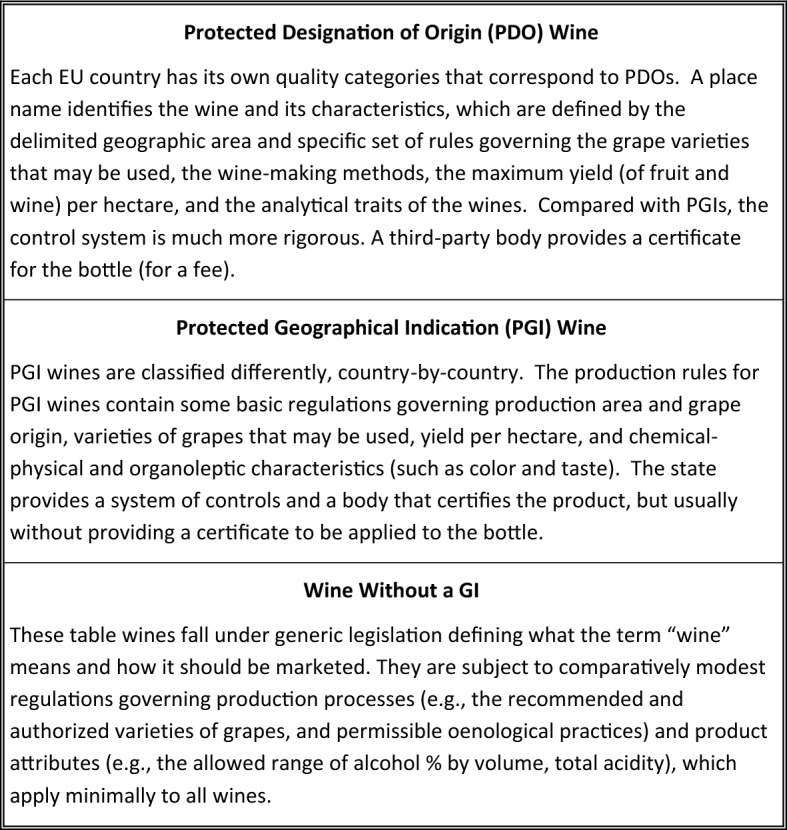
Heirarchy of European wine appellations

The EU wine law was conceived based on a quality pyramid, or hierarchy, with details of the structure of the pyramid varying among member countries and changing over time.^5^ In the current EU system of GIs, formed in 2008 and preserved in the 2013 CAP reform, wines are divided into two main categories: wines with a GI (i.e., appellation wine or quality wine), and wines without a GI (i.e., table wine). Appellation wines are divided into two sub-categories: Protected Geographical Indication (PGI) wines and, above that, Protected Designation of Origin (PDO) wines.^6^ The European regulatory framework permits the use of different national acronyms to preserve the traditional nomenclature introduced by each Member State (such as DOC-DOCG and IGT in Italy or AOC in France) to represent these categories (Fig. 1).^7^

## The CMO for Wine and Wine Policy under a Single CMO: A Brief History

The Common Market Organization (CMO) for wine was established in 1970, based on foundations laid in regulations passed in 1962 and supported by a common Customs Tariff passed in 1959 (see, e.g., Munsie 2002).^8^ It entailed several main elements in addition to those directly entailed in the appellation system. First, it introduced restrictions on both new plantings and replacement plantings of vines for the entire European Economic Community (EEC, now EU). These restrictions were closely tied to the appellation system. New plantings in quality wine regions were restricted to “recommended” and “authorized” varieties; new plantings for undifferentiated table wines (accounting for some four-fifths of production at the time) were restricted to authorized varieties. France applied more restrictive national regulations, permitting only recommended varieties to be planted in AOC regions. Second, a binding minimum support price was established for table wine supported by government purchases with complementary instruments for disposing of the resulting government stocks. Third, the wine policy provided for obligatory distillation to be used as an element of the price support system. Importantly, table wines and quality wines were subject to separate regulations and, for three decades, the CMO for wine related mainly to table wine production.^9^ As described by Munsie (2002), responsibility for implementing the policies was devolved to a great extent to the Member States, especially with respect to the specific technological regulations related to quality wine (Reg. 817/70).

### Policy Reforms, 1976–2013

Over the decades since, the wine law has been revised in the context of broader changes in the CAP, with substantive changes in the regulations in 1976, 1987, 1999, 2008, and 2013.^10^ The changes in policy were mostly responding to problems of excess supply and the resulting budgetary cost, but they also reflected shifts in thinking and changing market and policy context (European Commission 2020a).^11^ Here, we provide a brief chronological overview of the evolving market situation and policy emphasis, including budgetary details, to serve as a context for the analysis of particular elements of the policy in the subsequent sections.

Table 1 reveals the shifting balance of budgeted expenditures totaling €54.2 billion over the years 1971–2023, averaging more than €1.0 billion per year. Corsinovi and Gaeta (2017) interpret these shifts in expenditure in terms of three phases of policy: (1) focused on price and income support and consequences for supply management and surplus disposal; (2) still focused on the supply side, but reflecting a shift in emphasis to increasing overall quality both to be more competitive globally and reduce the cost of supporting unprofitable table wines; (3) focused on demand enhancement through quality improvement and product promotion. See, also, European Commission (2020a).

**Table 1:** Budgeted expenditure under the CMO for wine, main categories 1971–2023

Policy category	1971–80	1981–90	1991–00	2001–08	2009–13	2014–18	2019–23	1971–2023
**(a) Budgeted expenditure (million €)**								
Market withdrawals	563.5	3494.6	2621.0	–	407.9	–	–	**7087.0**
Distillation interventions	546.0	6308.7	4074.4	2344.2	419.1	400.8	453.0	**14,546.0**
Aid for market regulation	464.8	3302.9	5466.3	1105.5	787.4	735.5	719.3	**12,581.7**
Grubbing-up & abandonment	–	214.4	2330.9	844.7	124.9	–	–	**3514.9**
Restructuring & reconversion	26.9	40.0	743.7	2110.9	2221.2	2656.2	2277.2	**10,076.2**
Investments & innovation	–	–	–	–	518.3	1201.5	1415.1	**3134.9**
Promotion in third countries	–	–	–	0.0	522.0	1094.1	1132.4	**2748.6**
Conjunctional measures	–	–	–	130.9	200.5	153.6	44.5	**529.4**
**Total**	**1601.2**	**13,360.6**	**15,236.3**	**6536.1**	**5201.3**	**6241.6**	**6041.5**	**54,218.6**
Total per year	160.1	1336.1	1523.6	817.0	1040.3	1248.3	1208.3	1023.0
**(b) Expenditure shares (%)**								
Market withdrawals	35.2	26.2	17.2	–	7.8	–	–	**13.1**
Distillation interventions	34.1	47.2	26.7	35.9	8.1	6.4	7.5	**26.8**
Aid for market regulation	29.0	24.7	35.9	16.9	15.1	11.8	11.9	**23.2**
Grubbing-up & abandonment	–	1.6	15.3	12.9	2.4	–	–	**6.5**
Restructuring & reconversion	1.7	0.3	4.9	32.3	42.7	42.6	37.7	**18.6**
Investments & innovation	–	–	–	–	10.0	19.2	23.4	**5.8**
Promotion in third countries	–	–	–	0.0	10.0	17.5	18.7	**5.1**
Conjunctional measures	–	–	–	2.0	3.9	2.5	0.7	**1.0**
**Total**	**100.0**	**100.0**	**100.0**	**100.0**	**100.0**	**100.0**	**100.0**	**100.0**

Source: Created by the authors drawing on Corsinovi and Gaeta (2017), European Commission (2004, 2020b), and European Court of Auditors (2012)

Categories of expenditure used here are based on those defined by European Commission (2020b) (see, also Pomarici and Sardone 2020). “Distillation interventions” include aid for distillation schemes, buying-in of alcohol from compulsory distillation, and by-product distillation; “aid for market regulation” includes EU aid for storage, export refunds, and expenditure on regulatory measures (including SPS), and other interventions; “restructuring & reconversion” includes replanting of vineyards for health or phytosanitary reasons; “conjunctional measures” include support for setting up mutual funds, harvest insurance, and green harvesting practices. Estimates of expenditures prior to 2001–2008 are based on Corsinovi and Gaeta (2017). Figures for 2001–2008 were derived by deducting measures for 2009 and 2010 (taken from European Commission 2020b) from the estimates for 2001–2010 reported by Gaeta and Corsinovi (2014)

Under the CMO for wine, a very short initial period of equilibrium in the wine market was followed first by a very marked increase in production against a relatively constant demand, and eventually, a sustained decline and a very noticeable qualitative change in demand from the 1980s. Initially, the CMO was very open, with no curbs on plantings and very few market regulation instruments, but in short order it had generated a serious structural surplus giving rise to several policy changes in the 1970s and 1980s.

Beginning with changes in 1976, the policy became very interventionist, seeking to reduce the surplus using tighter restrictions on plantings (Reg. 1162/76) and financial incentives for taking vineyards out of production (Reg. 1163/76), while at the same time encouraging an increase in the overall quality. The effects of these policies and yield-restricting rules for appellation wines notwithstanding, wine producers continued to generate significant surpluses. Faced with massive harvests in 1979 and 1980 and protests from French producers, distillation was introduced as a basic means of regulating the market and eliminating surpluses; hitherto it had been used as an exceptional measure. Total expenditures under the CMO for wine increased from just €1.6 billion in the first decade (1971–1980) to €12.7 billion in the second (1981–1990), averaging €1.3 billion per year, predominantly for distillation and other measures to reduce the surplus of table wine.

In 1987, the patchwork of regulations and amendments was revamped and consolidated. Reg. 822/87 contained many of the past provisions but sought to strengthen them in order to push yields down and quality up, while Reg. 823/87 focused on quality wines, containing many of the same provisions as Reg. 817/70. These two regulations (822/87 and 823/87) formed the backbone of the CMO for wine until the 1999 reform undertaken as part of Agenda 2000. Expenditure averaging more than €1.5 billion per year during the 1990s continued to emphasize instruments that would reduce the surplus and support producer prices and incomes, but with some shifting toward instruments that would reduce productive capacity and enhance quality.

The 1999 reform to the CMO for wine was relatively modest.^12^ Regulation 1493/99 replaced and consolidated the main wine policy regulations and their patchwork amendments; for the first time, a single regulation governing both quality wine and table wine. Changes made here sought to: (1) achieve a better balance between supply and demand on the Community market; (2) give producers the chance to bring production into line with a market demanding higher quality; and (3) allow the sector to become competitive in the long term—especially in the face of increased global competition under the GATT. All of this was to be accomplished by financing the restructuring of a large part of present vineyards (see European Commission 2020a, b). This 1999 reform proved insufficient to reduce wine surpluses and considerable sums still had to be spent on disposing of them. A new reform of the wine market was needed. Adding to the existing pressures for reform, EU enlargement had integrated several more wine-producing countries (Hungary, Slovakia and Slovenia in 2004 and Bulgaria and Romania in 2007).

Surpluses were addressed initially through subsidized distillation of wine (to make distillate for biofuel and other industrial use as well as for potable use) and market withdrawals. Expenditure on these instruments grew from €1.1 billion in 1971–1980 to €8.5 billion during 1981–1990, but then declined to €5.2 billion during 1991–2000 and subsequently continued to diminish in absolute terms and as a share of expenditure on wine industry support. The balance was shifting to other instruments. As Corsinovi and Gaeta (2017) document, the quantities of wine distilled represented 21% of total EU wine production in 1981–1994, 13% in 1994–2007, and 11% in 2007–2011, after which these measures were phased out. The more rapidly shrinking subsidy expenditures reflected a smaller rate of subsidy applied to a shrinking volume distilled under subsidy. Table 1 includes details on expenditure on various other policies, which were important at times. Notable among these are subsidies for concentrated grape must (significant outlays in the 1980s, 1990s, and 2000s) and for storage of wine as a means of smoothing out supply and prices between seasons (significant outlays in the 1980s and 1990s), which are combined with expenditures on export subsidies and other interventions under “Aid for market regulation.”^13^

Bolder reforms were adopted by the EU in 2008 (see European Commission 2020a and Pomarici and Sardone 2020). Significant changes were made to policy instruments and the emphasis among them, and in implementation. Several traditional market intervention measures were eliminated, others were consolidated, and new ones were introduced. Responsibility for the detailed implementation of specific measures was devolved to individual Member States, and traditional market measures were combined with structural measures financed by a national envelope. The emphasis of the expenditure shifted from measures that would mainly apply to table wine to measures that would encourage the production of quality wine but with the detail of the implementation varying considerably from country to country within the EU.

The 2008 wine policy reform anticipated the 2013 general CAP reform. These changes made it easy to include the wine policy crafted in 2008 within the single CMO implemented in 2013 under Reg. 1308/2013. Pomarici and Sardone (2020) document in detail the full provisions in that policy, which applies for the period 2014–2020, and budgeted amounts directed to each of five structural measures and three conjunctional measures, collectively amounting to something over €1.0 billion per year. As can be seen in Table 1, comparing the period since 2008 with the period before then, the balance of the expenditure shifted profoundly toward restructuring and conversion, investments and innovation, and promotion—activities that promote quality wine as opposed to activities that support table wine and manage surplus production and stocks.

This period saw changes in the system of planting rights, which had been in force in some form since 1970 (see Sect. 5 for details). In 2008, the EU ministers of agriculture adopted a proposal by the European Commission to liberalize the regime by 2018. However, this proposal was opposed and overturned. Current rules implemented in January 2016 extend the planting restrictions to 2030, with some significant changes from the system in force for 1983–2015. First, authorizations are granted for free but are now non-transferable. Second, new authorizations allow up to 1% per year growth of a Member State’s area under vines. Third, authorizations may now be used for vines without a GI.

## Efforts to Reduce Persistent Problems

Problems of surplus production of table wine have been a recurrent feature of the European wine industry, exacerbated by the incentives provided by the market intervention elements of the EU wine policy. Most of the expenditures in Table 1 relate to this issue. The market support policies exacerbated the problems of excess capacity in the industry by encouraging investment or slowing disinvestment in ways that contradicted longer-term trends in market demand. In particular, especially in the earlier period of the CMO, they encouraged production of lower-tier wine. Meanwhile, the structure of demand was changing: demand was shifting against lower-tier European wine.

As documented by Anderson et al. (2018) and Holmes and Anderson (2017), the demand for European wine has been shifting in three ways. First, with globalization and increases in average income, age, and health-consciousness of consumers, per capita consumption of alcohol has declined, and the mix has shifted away from wine and towards other forms of alcohol—especially in traditional wine-consuming countries like France, Italy, and Spain. Second, the mix of demand in Europe and elsewhere has shifted towards more expensive wine—a trend of premiumization. Third, New World producers have increased their production, competing with European wine especially in the lower-priced tier. These changes in demand facing the European wine industry called for adjustments in production, but these were slowed by EU policies that blunted the signals from the market, coupled with an inherently inelastic supply response in an industry based on perennial crop.

Eventually, the excess supply problem was addressed by a combination of policies that reduced excess capacity directly, along with both a progressive reduction of the support price for table wine and other policies to encourage higher-quality wine production. Considerable changes were wrought in the structure of European wine production, especially in the balance between appellation wine (PDO or PGI) and table wine. Table 2 includes details on the area of vineyard and on the production of wine for both PDO/PGI wine and table wine for four countries (Germany, Spain, France, and Italy), which together contributed the great bulk of European wine production, since 1985.

**Table 2:** Shifting structure of European wine production, 1985–2018

Type by country	Year						
	1985	1990	1995	2000	2005	2010	2018
(a) Vineyard area							
Total (thousand hectares of vineyard)							
Germany	100.6	102.4	105.7	104.7	102.0	97.0	100.2
Spain	–	1390.4	1154.0	1167.7	1136.4	852.6	814.0
France	942.0	889.1	865.8	907.0	897.1	785.7	764.0
Italy	942.7	892.7	823.9	908.0	718.9	663.0	615.0
Total	1985.3	3274.5	2949.5	3087.4	2854.4	2398.3	2293.1
PDO/PGI (thousand hectares of vineyard)							
Germany	100.6	102.4	105.7	104.7	102.0	96.8	100.0
Spain	–	667.3	596.8	619.8	656.4	525.1	505.0
France	493.5	515.4	522.1	536.2	543.0	674.4	663.1
Italy	198.7	160.3	183.1	242.9	286.0	320.9	444.7
Total	792.7	1445.3	1407.8	1503.7	1587.4	1617.1	1712.8
Table wine (thousand hectares of vineyard)							
Germany	–	–	–	0.0	0.0	0.2	0.2
Spain	–	723.1	557.2	547.9	480.0	307.5	289.1
France	446.6	373.7	343.8	334.2	314.4	105.1	93.7
Italy	744.0	732.4	652.3	481.9	432.9	304.8	125.3
Total	1190.6	1829.2	1553.2	1364.0	1227.3	717.6	508.4
% PDO/PGI (percent)							
Germany	100.0	100.0	100.0	100.0	100.0	99.8	99.8
Spain	–	48.0	51.7	53.1	57.8	61.6	62.0
France	52.4	58.0	60.3	59.1	60.5	85.8	86.8
Italy	21.1	18.0	22.2	26.8	39.8	48.4	72.3
Total	39.9	44.1	47.7	48.7	55.6	67.4	74.7

Type by country	Year						
	1985	1990	1995	2000	2005	2010	2018
(b) Wine production							
Total (thousand hectolitres)							
Germany	5.4	8.5	8.9	10.1	9.1	7.0	10.3
Spain	–	42.2	22.7	46.1	41.1	40.7	49.6
France	69.2	65.5	55.6	59.8	53.4	46.5	49.6
Italy	62.3	54.9	59.3	54.1	50.6	50.6	55.8
Total	137.0	171.1	146.5	170.1	154.2	144.8	165.2
PDO/PGI (thousand hectolitres)							
Germany	5.4	8.5	8.9	10.1	9.1	6.9	9.6
Spain	–	20.5	13.3	25.5	12.1	17.5	21.4
France	19.3	23.6	24.6	36.0	34.3	36.1	36.9
Italy	7.9	8.8	9.8	14.9	15.0	31.3	35.9
Total	32.5	61.5	56.6	86.5	70.5	91.8	103.8
Table wine (thousand hectolitres)							
Germany	–	–	–	–	0.0	0.1	0.1
Spain	–	21.7	9.4	20.7	29.0	21.3	23.4
France	50.0	41.9	31.0	23.8	19.2	10.1	12.7
Italy	54.5	46.1	49.5	39.2	35.5	19.1	17.9
Total	104.5	109.6	89.9	83.6	83.8	50.6	54.0
%PDO/PGI (percent)							
Germany	100.0	100.0	100.0	100.0	100.0	98.7	93.6
Spain	–	48.7	58.8	55.2	29.4	42.9	43.1
France	27.8	36.1	44.2	60.2	64.1	77.7	74.4
Italy	12.6	16.1	16.5	27.6	29.7	61.9	64.4
Total	23.7	35.9	38.7	50.9	45.7	63.4	62.8

Source: Created by the authors drawing on various online sources including: http://appsso.eurostat.ec.europa.eu/nui/show.do?dataset=vit_an7&lang=en, https://appsso.eurostat.ec.europa.eu/nui/show.do?dataset=vit_an5&lang=en, http://appsso.eurostat.ec.europa.eu/nui/show.do?dataset=ef_lpc_vineyd&lang=en, https://ec.europa.eu/agriculture/sites/agriculture/files/wine/statistics/harvest-forecast-2018-2019_en.pdf, https://ec.europa.eu/agriculture/sites/agriculture/files/wine/statistics/historic-market-situation_fr.pdf, http://seriestoriche.istat.it/index.php?id=1&no_cache=1&tx_usercento_centofe%5Bcategoria%5D=13&tx_usercento_centofe%5Baction%5D=show&tx_usercento_centofe%5Bcontroller%5D=Categoria&cHash=e3503d8195dd4231ff53ba078ad5c124

In 1990, after 20 years under the CMO, the four countries (Table 2, panel a) together had 3.3 million ha of wine grapes of which 44.1% was appellation wine; the share of production (Table 2, panel b) was lower (35.9%), reflecting the higher yield for table wines. During the 1980s and 1990s, the CMO interventions continued to support table wine, and its share of the total area and volume of production remained high. But after the turn of the century, the structure of production shifted much more rapidly, reflecting a shift in policy seeking both to reduce the total area of vineyard and to increase the quality wine share in that total.

By 2018, the total the area of vineyard had fallen by almost 1 million ha (30%) from the peak in 1990, with most of the reduction accomplished by 2010 (Table 2, panel a). Production fell by a smaller proportion, reflecting increases in average yields as poorly producing vineyards were being grubbed out (Table 2, panel b). The share of appellation wine had increased to almost 50% of the total area and more than 50% of the total volume by 2000, and by 2018 it was up to 75% of the area and over 60% of the volume. However, some of this change reflected the fact that the new 2008 regulation restructured the categories and changed the definition of appellation wines. Certain table wines were simply elevated to the rank of PGI wines, without any change in their quality.

In spite of the efforts over many years to improve the balance of supply and demand, a significant structural surplus remains, especially for table wine but now also for some appellation wine. In 2019, the EU spent a total of €1.2 billion supporting the wine industry, one way or another. The long-term structural problem of excess capacity in the EU was exacerbated by a depressed global wine market. These difficulties were compounded for the EU industry by trade disputes with the United States, manifest as tariffs on imports of wine from the EU.^14^ Adding to these woes, in early 2020, the COVID-19 pandemic devastated the wine industry worldwide, by causing closure of restaurants and bars and winery tasting rooms.^15^

## Supply Management Policies

Various supply management policies were introduced at different times to mitigate the consequences of excess capacity and surplus production in the European wine industry, including subsidized distillation of wine, other supply diversion instruments, subsidies for grubbing out vineyards, and planting restrictions. Among these instruments, planting restrictions are the most intrusive and most important. Indeed, as Meloni et al. (2019, p. 639) say, given the size and significance of the EU wine industry, “…the European system of vineyard planting restrictions is one of the most important policies affecting global wine markets today.”

Problems of excess supply became apparent early on. Planting rights were introduced at the outset, in 1970—limiting the total area of vineyards by regulating new plantings.^16^ Since then, policies restricting new plantings have remained in force, with some variation in the details over time including some significant changes in 2016, as noted above (see, e.g., Meloni et al. 2019; Pomarici and Sardone 2020). Deconinck and Swinnen (2015) present a detailed and elegant analysis of the economic consequences of this complex policy, which created various rights (replanting rights; rights for planting from a reserve; and new planting rights) that were to some extent transferable among producers and places. In their base analysis, Deconinck and Swinnen (2015) assume perfect enforcement of a freely transferable production right—treating an area right as effectively a right to produce and sell winegrapes and wine. In subsequent sections, they explore consequences of restrictions on transfers, reserves, and imperfect enforcement of these rights. Throughout their analysis, however, Deconinck and Swinnen (2015) treat land as the only input and assume uniform, exogenous quality. This assumption is open to challenge, and it matters.

Previous studies have shown that producers will respond to area restrictions by intensifying the use of other inputs per unit area, which has the effect of increasing yield and (typically) reducing intrinsic output quality; conversely, producers will respond to output restrictions by managing production to increase quality, at the expense of yield (e.g., see Foster and Babcock 1990, 1993; Alston and James 2002; James and Alston 2002). Planting rights for wine grapes might have entailed some such effects. In the case of low-quality (table) wine, the EU area restrictions did not also entail yield restrictions, so producers had an incentive to intensify use of variable inputs and yield, even if it resulted in lower quality. In contrast, in the case of appellation wines, if maximum yield rules were already binding, the addition of area restrictions implied an output restriction of the type assumed by Deconinck and Swinnen (2015). However, if other inputs are variable and producers can profitably vary input proportions, an output restriction should induce an increase in quality. In other words, in the context of the European wine markets, we expect planting restrictions induced an increase in average quality for appellation wines, a decrease in average quality for table wines, and an increase in the quantity of low- relative to high-quality wines. The ultimate impacts in such a case depend on the extent to which rights are transferable between high- and low-quality production systems, and on the role of other interventions.

In parallel with the declining expenditure on distillation subsidies since 1990, expenditure increased on subsidies for grubbing up or abandonment of vineyards (reducing total capacity), for restructuring and reconversion of vineyards (shifting from low- to higher-quality production), and green harvesting (removing 100% of fruit from selected vineyards to reduce supply in the current vintage). Expenditure on these instruments grew from €0.3 billion during 1981–1990, to €3.1 billion during 1991–2000, €3.8 billion during 2001–2010, and €3.8 billion during the 5 years, 2011–2015, with the balance shifting over time from grubbing up to green harvesting and, more importantly restructuring and reconversion of vineyards. Subsidies for restructuring and reconversion of vineyards contributed to a significant reduction in excess capacity, especially at the troublesome low-quality end of the spectrum, contributing to premiumization in terms of the varietal mix and location of production within and among the main Member States. Over the period 2009–2023, restructuring and reconversion was the largest line item, accounting for 41.1 percent of budgeted expenditure on wine under the CAP (European Commission 2020b).

As the European Commission reports (2004, p. 65): “… during the first period (1977–1984), yield was on average increasing [by] about 0.9 HL/ha annually. During the second period (1985–1996), yield decreased by about 0.1 HL/ha per year. Between 1996 and 2000 yields annually increase about 0.66 HL/ha. … This indicates that yields seem to have been influenced by the CMO instruments (compulsory distillation, which discouraged high yields, and the premium for permanent abandonment which reduced the area of high yield, low-quality vineyards).”^17^ The report describes specifically how the changes in rules over time coincided with the changes in the path of yields.

## Economic Consequences of GIs for European Wine

The pre-existing appellation law in France contributed substantively to the design of the counterparts in the CMO for wine established in 1970, with core elements carrying forward to the present day. When the AOC policy was introduced in 1935, it combined two government purposes: (1) overcoming the lemons problem and (2) reducing total supply, especially of low quality wine.^18^ First, the lemons problem was to be addressed indirectly, by regulating the process of production (varieties, yields, winemaking practice) for wines that could be labeled as coming from a particular AOC, adding to the signal of quality associated with the place (i.e., the GI, alone). The resulting premia for wines from within the AOCs made it attractive for eligible producers to comply with the requirements, and for other producers to strive to establish their eligibility; the rights, attached to land within particular AOCs, became valuable almost immediately. Second, given the participation of eligible producers, the regulation of their yields reduced supply directly.

These two main effects together led to improvements in profitability for growers, and probably benefited consumers as well—although consumers must be made worse off by an increase in price associated with supply restriction per se, the “cartel” aspect of the policy. Mérel et al. (2019, p. 3) concluded that the “market price of appellation wine in France increased significantly due to the recognition of AOC vineyards, by a value roughly equal to 45% of the average price of wine.” Applying that price increase to the 30.5% of French vineyards eligible for AOC recognition implies a very substantial gross welfare gain (on the order of 14% of the gross value of production), but this does not account for the added costs of “…quality-enhancing practices required for wines sold under the AOC label” (Mérel et al. 2019, p. 4).

Today’s EU wine law contains corresponding elements that purport to achieve similar purposes related to signaling quality and managing total supply. However, the market situation is different in several important ways, making it less likely that the policy is beneficial to society as a whole; to be sure, some consumers and some producers are made worse off. To analyze the economic consequences of the EU wine policy it is helpful to tease out its two main elements: first, the creation of GIs, which can be used to convey signals of quality and create a collective reputation associated with a place per se (or perhaps its terroir); and second, technological regulations: restrictions on the production process for wine to be designated as coming from a particular GI.^19^

### Geographical Indications as Collective Brands

GIs enable the creation of collective brands associated with particular production regions.^20^ In principle, by defining the production process, the regulations provide some assurance of product quality attributes, thereby facilitating the creation of a collective reputation (Winfree and McCluskey 2005).^21^ This collective reputation can be an extremely valuable asset to individual producers, as reflected in land values. The word Champagne, for example, refers to sparkling wine from the Champagne region produced according to the rules for that appellation. The collective brand Champagne therefore connotes a product defined in terms of both the place where it was produced (including the terroir) and the process of production. It conflates the place and the technological regulations.

It is not a simple matter to pull these elements apart, though we can readily imagine a scenario in which currently disallowed grape varieties or production practices might be profitably employed by a Champagne house, nowadays, without harming quality or the collective reputation of the region, if it would be allowed. Collective brand-names like Champagne are jealously protected because they serve as a barrier to entry that prevents free-riding. If a comparable wine is produced by the same process in some other region, such as Franciacorta, it is not allowed to be called Champagne.^22^ Under the umbrella of the collective brand-name for the appellation, individual wineries may produce a diverse range of wines that they can sell under their own individual brand name(s) or trademarks. One open question is the extent to which individual brands can be seen as complements to or substitutes for the collective brand-name of the appellation (see, e.g., Menapace and Moschini 2012).^23^

In other countries, regional appellations are also used widely for wines, in many cases without any comparable technological regulations. This provides a basis for comparison from which we might draw inferences about the potential consequences if European governments were to eliminate process regulation, while preserving the other GI aspects of the EU policy. The United States offers an informative example. As described by Lapsley et al. (2019), in 1980 the U.S. Government created its counterpart to PDOs, American Viticultural Areas (AVAs—see U.S. Treasury/TTB, 2013).^24^ In 2019, the United States had a total of 245 established AVAs. California, which produces about 90% of U.S. wine, has 139 AVAs of which 16 are located in the Napa Valley AVA (see U.S. Treasury/TTB 2018).^25^ Prices of wine and the grapes used to produce it vary considerably among AVAs, even within the state of California, which includes diverse wine regions (see, e.g., Alston et al. 2015).

Even though the AVA rules do not prescribe maximum yields or varieties, producers in California are known to manage their yields with a view to enhancing quality, and some wineries market their wines as produced from low-yielding vines. In practice, a relatively small range of varieties predominates, chosen by growers to match their terroir and the market (see Alston et al. 2015). Consequently, even without any regulation requiring it, varieties tend to be associated with AVAs—such as Cabernet Sauvignon with the Napa Valley AVA and its sub-appellations, and Pinot Noir with the Willamette Valley AVA and its sub-appellations in Oregon as well as the Carneros AVA in California. Indeed, as shown in Table 3, even though the Napa Valley AVA includes part of the Carneros AVA, which is more like Burgundy, the mix of varieties grown in the Napa Valley AVA corresponds closely to the mix grown in Bordeaux (Gironde). In both places, a few varieties dominate. Just six varieties (Cabernet Sauvignon, Cabernet Franc, Chardonnay, Merlot, Sauvignon Blanc, and Semillon) account for 96% of production in Bordeaux; the same six varieties account for almost 80% of production in the Napa Valley even though producers could freely choose to plant many others. Moreover, as discussed by Alston et al. (2015), the main premium wine producing regions of California, Oregon, and Washington State grow a mix of wine grape varieties that is very similar to the mix in France, and becoming more so over time.^26^ As discussed by Anderson (2014), this is a more widespread global phenomenon.

**Table 3:** Comparing Napa and Bordeaux: area of vineyard for top 10 varieties in 2017–2018

Bordeaux (Gironde)			Napa county		
Variety	Area (Ha)	Share	Variety	Area (Ha)	Share
Merlot	71,637	58.5	Cabernet Sauv	8801	46.5
Cabernet Sauv	2901	19.5	Merlot	1738	9.2
Cabernet Franc	9712	7.9	Other reds	1340	7.1
Cot/Malbec	1811	1.5	Pinot noir	1142	6.0
Petit verdot	1094	0.9	Zinfandel	523	2.8
Other reds	655	0.5	Cabernet Franc	489	2.6
			Petite sirah	351	1.8
Semillon	6433	5.3	Petit verdot	321	1.7
Sauvignon blanc	5934	4.9	Syrah	279	1.5
Muscadelle	750	0.6			
Colombard	271	0.2	Chardonnay	2481	13.1
Chardonnay	127	0.1	Sauvignon blanc	1138	6.0
Other whites	56	0.1	Other whites	316	1.7
Total	122,381	100.0	Total	18,917	100.0

Source: Created by the authors drawing on the 2018 Agricultural Commissioner’s Report for Napa County. Available at https://www.countyofnapa.org/DocumentCenter/View/13095/2018-Napa-Crop-report-English-Version-?bidId=, and France AgriMer Stats 2017. *Les chiffres de la filière viti-vinicole, Données statistiques 2007/2017*, 2018. Available at https://www.franceagrimer.fr/content/download/59141/920125/file/chiffres-fili%C3%A8re-viti-vinicole-2007-2017.pdf

### Too Many Appellations?

As in Europe, we can observe a hierarchy of sub-appellations in other countries, with a tendency for the broadest appellation to be associated with the lowest-priced wines, and for prices to increase as the (sub-)appellation becomes narrower, and more-specific (if it were not so, the winery would choose not to use the narrower, more-specific designation).^27^ Since more-specific appellations tend to signal higher-priced wines, wineries tend to prefer, where possible, to use narrower designations resulting in a greater number of smaller GIs—possibly beyond that implied by heterogeneous terroir alone.

This tendency might have contributed to the phenomenon reported by Livat et al. (2018) in the case of Bordeaux: we can have too many appellations, in the sense that they no longer serve as an effective signal of quality for consumers. The situation can be seen as a kind of over-fishing problem if it is profitable for a particular group of producers to create an incremental appellation (or sub-appellation) but not for society as a whole. The overfishing problem arises if this behavior leads to an excessive number of appellations, creating the type of information problem the policy was meant to solve.^28^

A further potential source of confusion is the changing geographical scope and nature of some appellations. Champagne and Prosecco provide interesting examples of adaptation at work. In the case of Champagne, reflecting the fact that the boundaries of appellations are somewhat arbitrary, the area of the denomination has been increasing—it grew from 30,900 ha in 2007 to 33,800 ha in 2017.^29^ In the case of Prosecco, the redefinition of the appellation in the same time frame was much more radical, with three major changes introduced in 2009.^30^ In the years since, the world has witnessed great growth in production and export of Prosecco wine (made by the méthode charmat), especially to the UK market, where it has substantially displaced Spanish Cava (made by the more expensive méthode champenoise).^31^

## Technological Regulation

Clearly, wine producers worldwide see virtue in establishing GIs as a way of creating a collective brand associated with a particular place of production. At issue is the extent to which the technological regulations in the GIs for European wine contribute to the value of the wine, by enhancing quality and signaling quality in ways that are valuable for producers, consumers, and the society as a whole. The answer to this question may have changed over time.

In 1935 when the AOC system was first introduced in France, the situation in the wine industry was quite chaotic in a market flooded with poor-quality wines produced with ill-favored varieties. The designers of the AOC system identified the best varieties for each region and production practices that would contribute to making better wines; to a great extent these were the major producers in each AOC, codifying their established practices. By promoting the adoption of these varieties and practices, the introduction of the AOC system eventually helped to reduce the wine glut, to improve wine quality for producers who chose to participate, and to reduce the lemons problem.^32^

A more subtle and longer-term consequence is that, at least for a time, the strictures over eligible production practices and processes promoted learning-by-doing and a culture of quality management in the industry, both in grape-growing and vinification. In addition, consumers were able to become better educated about wine quality through increased availability of more differentiated wine. These didactic aspects were a direct consequence of the regulations but they also enhanced other benefits by encouraging participation. Similarly, the introduction of appellation wine rules had a mixture of consequences in the 1960s and 1970s in Italy, Spain, and Portugal, including didactic elements. At issue is whether the current set of process regulations continues to yield benefits of this nature. At least some of the benefits from inducing a transformation of the industry and its culture, reducing the lemons problem, and facilitating consumers and producers to be better educated about wine, were of a one-time, transitory nature. And some may have been continuing but were reduced or eliminated by other changes. A case in point is varietal regulations.

### Varietal Regulations

Unlike California’s AVAs, European PDOs for wine designate the grape varieties that may be used to produce PDO wine; and in some cases, the minimum and maximum proportions of each of those varieties in the blend. Producers in the same region are free to grow other varieties, which may be eligible for labeling as PGI wine, but they must satisfy varietal blend restrictions if they wish to use the valuable PDO designation. This can be a strong incentive to grow only those designated varieties.

Nowadays, however, many wines are sold with information about the varietal content of the wine on the label. This is particularly so for premium wines produced in the New World, but also increasingly so in Europe—and not only wines classified as “varietal wines” within the EU classification, which are non-GI wines. If varietal information is (or can be) provided on the label, what additional information is conveyed by a law that restricts those varieties to come from a particular short list or in a particular mixture? Today, the term Bordeaux conveys both the region and the potential varietal blend. But in principle these aspects could be distinguished if both a traditional Bordeaux blend and other blends were permitted to be produced from within the same delimited PDO region and allowed to use the same PDO designation. Otherwise, denying the use of the designation Bordeaux is an implicit signal that the non-traditional blend is in some sense inferior—if only because it is non-traditional.

The implicit stigma against nontraditional varieties might be costly. Several aspects of the environmental and market context are placing pressures on the wine industry that potentially could be addressed by genetic innovations in varieties (either in the scions or the rootstocks to which they are grafted). Evolving consumer demand is one set of forces driving the demand for different types of grapes with different bundles of traits, including agronomic traits that facilitate the use of production processes that qualify for eco-labels such as organic, biodynamic, sustainable, or non-GMO. Another set of forces is the public policy processes that are applying increasingly stringent restrictions on the use of pesticides and other agricultural chemicals in vineyards, increasing the demand for alternatives, including pest- and disease-resistant varieties. Finally, climate is changing in ways that have implications for the compatibility of varieties with the locations where they have been traditionally grown (see, e.g., Jones 2006, 2016, 2018).

Existing PDO rules constrain the potential for varietal innovation to address these stresses. But the varietal name per se is an important and valuable attribute of existing varieties used for wine making, even in the absence of PDO rules that prescribe growing traditional varieties. Varietal innovation will be hampered if the value of varietal names is large relative to the value of other traits that might also be desired such as higher yield, resistance to pests and diseases, or fruit quality attributes. Nevertheless, some European producers have found it profitable to switch from PDO to PGI in order to be able to make varietal and other innovations (not admitted in PDOs).^33^ In the case of Super Tuscan wines, the upshot was an upside-down pyramid, with higher prices for PGI than PDO wines.

Changes in climate and consumer demand for eco-labels may lead to further movement in this direction and ultimately might induce changes in the policy. For example, in 2019, producers in Bordeaux voted to allow the use of seven new varieties for production of Bordeaux Supérieur wines (i.e., within the PDO, but not for the sub-appellations such as Pauillac or Saint-Émilion). These varieties are all *V. vinifera*, including some that are commercially significant in other parts of Europe, some that are crosses between familiar *vinifera* varieties, and some that are little known. These varieties might be adopted in response to changes in climate. Hybrid varieties offer more hope for coping with pests and diseases, and we see changes in this area, too.^34^

Environmental, health and cost concerns are leading European producers and policymakers to reconsider hybrids, which could allow for significant savings in costs to growers and reduced environmental and human health risks (see, e.g., Sambucci et al., 2019). In response to pressure from the industry, revisions introduced in the 2009 CMO permit the use of hybrids (crosses with *V. vinifera*) to produce PGI wines, while preserving the rule that only *V. vinifera* varieties may be used to produce PDO wines. Grape breeders have succeeded in developing *vinifera*-like varieties with desirable pest- and disease-resistance attributes, and already in 2017, new hybrid varieties have been released by the French National Research Institute (INRA).

With a view to improved resistance to pests and diseases and resilience to climate change, the 2018 CAP reform proposal provides for a wider use of intra- and interspecific hybrids in both PDO and PGI wines (see, e.g., Pomarici and Sardone 2020). However, individual Member States retain control over which varieties may be planted within individual PDOs and PGIs, depending on what their producers may wish. Moreover, even if permitted, growers might not find it profitable to adopt these hybrids if the hybrid varietal discount exceeds the fungicide cost savings (see, e.g., Fuller et al. 2015). These tradeoffs may become more favorable in the future, but for now this change in the rules does not promise to lead to major changes in practice any time soon.

Then we have genetic engineering. To a modern geneticist, cross-breeding (or random mutagenesis) and selection is a clumsy and imprecise way of improving varieties compared with gene-editing (drawing on the plant’s own genes to manifest new traits) and the related tools of genetic engineering (transgenic modifications, producing GMOs). The rise of widespread opposition to GMOs in Europe was to a great extent driven by NGOs spreading false propaganda (see, e.g., Lynas 2018), and is based largely on uninformed perceptions about the environmental consequences of GMOs or the human health risks they pose: GMOs are often safer for people and the planet than the conventional technologies they would replace (Qaim 2009; Klümper and Qaim 2014).^35^

Notwithstanding the mountain of scientific evidence, including that produced by European authorities, showing that GMOs pose no greater risk than conventional technologies, they have been effectively blocked and are not used to any meaningful extent in Europe. If that were not bad enough, unfortunately, the relevant European authorities appear to have determined that new breeding techniques (NBTs) like gene-editing should be regulated like GMOs. In contrast, the USDA (which regulates GMOs) does not plan to regulate plants that were developed using NBTs if they could otherwise have been developed through conventional breeding. The upshot is that the European wine industry will have a much more limited range of options available as it seeks to respond through genetic innovation to the diverse pressures coming from the shifting environmental and market context. This is not a uniquely European problem. Even in the United States where GMOs have relatively high acceptance, the wine industry is cautious about GMOs and many consumers are demanding wines with ecolabels favoring traditional technologies (Box 1).

**Table:** 

**Box 1: Ecolabels and Other Label Claims**
In some senses the demand for ecolabels reflects a preference for anything but genetic innovations, even though genetic innovation—through hybrids or gene-editing—might have the greatest potential for providing sustainable solutions to environmental threats such as climate change and evolving pest and disease pressures. Around the world, led from Europe, consumers and environmental activists are demanding a shift toward more environmentally sustainable production systems throughout agriculture, including vineyards and wine. Many wine producers themselves share these aspirations, even to the extent of adopting practices that would be unprofitable otherwise.
Consequently, for both ethical and commercial reasons, a growing share of the total volume of wine is produced using various purportedly sustainable practices, and some of that is sold with some kind of ecolabel—such as organic, biodynamic, natural, sustainable, or non-GMO. Still, even in France only 10% of the total vineyard area in 2017 was organic (Alonso Ugaglia et al. 2019). In some cases, like organic, these terms are clearly defined and have legal meaning (supported by government certification), though the meaning may vary among jurisdictions. (For example, in the United States organic wine means wine made with organically grown grapes and without added sulfites, but in the EU sulfites can be added lawfully in making organic wine.) This is a profoundly important difference because making stable wines without sulfites is much more difficult. In other cases, such as “natural,” the terms are used much less formally, without any specific legal definition. The term biodynamic falls in between, using private certification with a defined set of rules, networked internationally through Demeter International.
One problem with these terms is that most consumers are not well-informed about the specific meaning and implications of particular ecolabels. Uninformed consumers might presume that an ecolabel carries with it the promise that the grapes were produced without using synthetic pesticides such that the wine might have desired food safety attributes and a smaller environmental footprint. Even if this were true for organic wine, which is open to challenge, the extent of the benefits is not easy to judge; even more so with other ecolabels like natural or sustainable, when promises regarding production practices are vague. Consequently, we face a new lemons problem in the context of asymmetric information about the environmental footprint and food-safety aspects of wine conveyed by ecolabels. The commercial use of such terms has not been much regulated, an example of government failure.

### Yield Restrictions

Both PGIs and PDOs typically specify maximum yields per hectare in terms of both fruit production (kg/ha) and wine production (hl/ha). These restrictions are supposed (commonly claimed) to have the effect of improving the quality of the wine, but even this is a somewhat controversial idea. To be sure, the introduction of process regulations as part of appellation rules did lead to substantial improvements in average quality in the early years (see, e.g., Mérel et al. 2019); and in today’s market, across locations, and across vintages for a given location, higher yields tend to be associated with lower-priced wine grapes and lower-priced (or lower-rated) wine (see, e.g., Alston et al. 2015). Even so, in many cases in today’s market, these yield restrictions that are very costly for producers could be relaxed without causing a material loss in wine quality (i.e., in the average revenue per unit holding quantity constant).

Yield restrictions are controversial for two reasons. First, among grape growers and scientists working in the area, there is some debate over whether cultural practices that reduce yield are an effective or economical means for improving quality of the vintage compared with other management practices (see, e.g., Matthews 2015). It is a complex question because quality has many dimensions, and growers have many means available for managing yield and fruit quality attributes.^36^ The links between these management practices, as they affect the total yield, and diverse fruit attributes that can contribute to various aspects of wine quality are complicated and not always well-understood, even by grape growers and other experts.

At least over some of the feasible yield range we would expect to observe a trade-off between yield and quality—at a minimum this can be accomplished by grading out lower quality fruit and wine—and it is likely to be economically rational for producers to be producing within that range if they are free to do so. But as we show next, it is much less likely to be economically rational for society to restrict the producers’ choice within that range using a blunt instrument policy of yield restriction applied uniformly to all producers within the appellation.

**Fig. 2:**
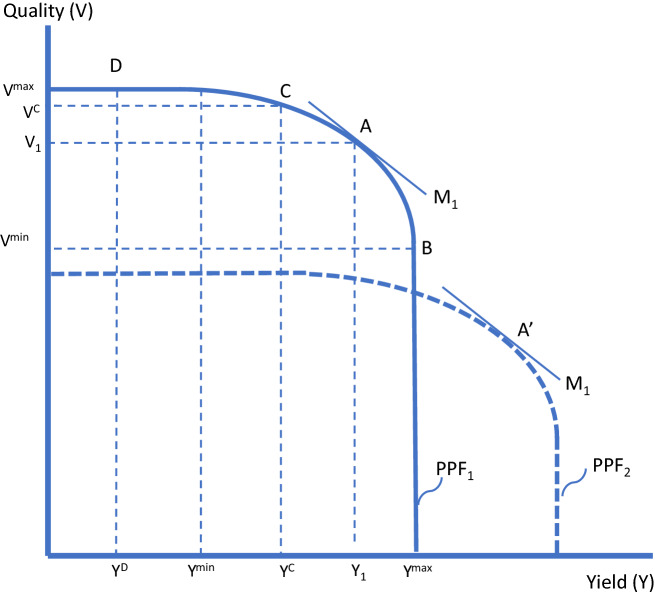
Quality consequences of yield restrictions

To begin, consider an appellation that includes numerous identical producers, each of which is producing at some point on the same quantity-quality production possibilities frontier as depicted in Fig. 2 for a representative hectare: labeled PPF_1_. In this figure, V^min^ denotes the minimum (economic) quality in the sense that further reductions in quality do not allow further increases in yield (above Y^max^), while V^max^ denotes the maximum (economic) quality in the sense that further reductions in yield (below Y^min^) do not allow further increases in quality. If markets were functioning well at signaling the demand for wine quality attributes, producers would all choose to produce at point A, where the magnitude of the slope of the production possibilities frontier is equal to the quality premium (indicated by the line, M_1_). At this point, yield and quality are optimal at Y_1_ and V_1_. However, if consumers cannot discern quality, such that the premium for quality is zero, producers would be induced to produce at point B (the maximum quantity)—an extreme lemons problem, with quality, V^min^.^37^

Suppose a social planner imposes a maximum yield, Y^C^ aiming to correct the distortion. In this scenario the constraint will be exactly binding: the regulated maximum yield will become the yield, with its associated quality V^C^—i.e., point C. The challenge for the social planner is to understand enough about the trade-off to set the constraint in the right place—even in this simplest possible scenario. In reality, this is a very serious challenge. In an extreme case, the social planner might set a maximum below the yield, Y^D^ that corresponds to the maximum possible quality, V^max^ such that over some of the range, yield and output could be increased without any reduction in quality. We have informal evidence—based on discussions with numerous grape growers and wine makers about their personal experience, and broader observations from scientists and others—that at least some and perhaps many producers are restricted to this extent by current rules. They could significantly increase their yield without any loss of quality.

A second set of issues arises when the same yield restriction is imposed across places with diverse production environments and across varied seasons in a given place. A restriction that is close to the median producer’s optimum quality-quantity tradeoff, will not be binding for half the producers and will be overly restrictive for the other half. To illustrate, Fig. 2 includes a second production possibilities frontier, labeled PPF_2_, with the two frontiers potentially representing different producers (producers 1 and 2) in the same year, or the same producer in different years (vintages 1 and 2). It is easy to see in this figure how a yield restriction at Y_1_ that is optimal for producer 1, given the market valuation of quality, M_1_, is unlikely to be optimal for producer 2 or for producers as a group. This is a significant concern since, even within a modest-sized estate of a few hectares, we might find individual patches with very different soil types, terrain, or exposure, which taken together may imply very different production possibilities (i.e., terroir).

Likewise, for any given producer, when seasonal conditions vary across vintages in ways that shift the quality-quantity tradeoff—which they surely do—a yield restriction that is optimal for vintage 1, at Y_1_ given the market valuation of quality, M_1_, will not be optimal for vintage 2. This observation, related to the effects of year-to-year weather changes also applies to secular changes in the production possibilities frontier associated with improved understanding of viticulture and enology, the adoption of alternative production systems, or climate change. This can be important if yield restrictions determined at a particular point in time might be held fixed over many years or even decades.

A third set of issues arises because the social planner is presumed to know the optimal premium for quality, the associated optimal quality mix, and the (maximum) yield that will result in that perfect outcome. This is a lot to know, even if the relationships would be fixed over time and among producers and if quality were a simple, one-dimensional construct, as it has been presented in this didactic exercise. In fact, wine is one of the world’s most highly differentiated products, its quality is multidimensional, and the market is constantly in flux. The potential for error is large. What if the relevant price premium is very different from M_1_, or if the relevant premium is very different among different producers or must change because the market has evolved?

## Technological Regulations in a Market Context

Yield restrictions ostensibly serving as quality assurance might also have been intended to limit production and raise prices and profits for producers. While potentially increasing average quality, binding yield restrictions also have the effect of reducing the volume of appellation wine produced, which in turn has consequences for prices, production, and consumption of wine. The ultimate consequences in reality will depend on how well the policies are enforced, which will depend on the costs of enforcement and cheating.

### Market Effects of Yield Restrictions

**Fig. 3:**
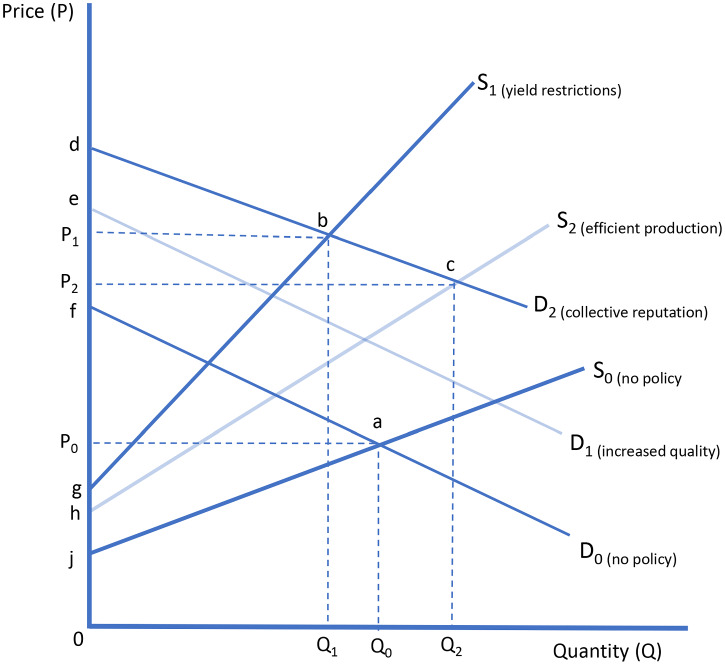
Market effects of yield restrictions in PDOs

In Fig. 3, S_0_ is the total supply of wine from a specific region in the absence of yield restrictions or other controls, and D_0_ is the demand for wine from the region, which slopes down—the region is large in the sense of being able to influence the price of its wine by managing its supply. Certainly, Europe is a large producer and exporter of both quality wine and table wine, in this sense, as is each of the main wine producing countries: France, Italy, and Spain. It can be profitable for the industry in a large country or region to restrict supply and exercise market power in trade at the expense of both foreign and domestic consumers, and the resulting gains to domestic producers might even exceed the costs borne by domestic consumers. However, even the largest wine GIs would not be expected to have much market power in the sense that, if they were to independently restrict their total supply, they could generate an appreciable increase in their average revenue or price (which requires downward sloping demand facing the GI) let alone total revenue (which requires inelastic demand facing the GI). Overall demand is likely to be fairly elastic even for the largest and most distinctive wine GIs. Moreover, yield restrictions have serious deficiencies as instruments of supply control, and at least some of the potential gains from the exercise of market power will be dissipated in costs of input mix distortions, as we illustrate next.

Let us assume that, when the region establishes a PDO or PGI, with restrictions on yield and other aspects of production, this results in an increase in average quality.^38^ In Fig. 3, the increase in quality is represented as an increase in demand from D_0_ to D_1_ (reflecting an increase in consumer willingness to pay for any given quantity). A further increase in demand, from D_1_ to D_2_ is implied if, at the same time, the policy has resulted in an improvement in consumer confidence in the quality of the product, a collective reputation effect. Meanwhile, the industry supply (and marginal cost) function shifts up to the left, from S_0_ to S_1_ reflecting the increased cost of production caused by the restrictions.

Consequently, the market equilibrium has shifted from the intersection of D_0_ with S_0_ at point a (Q_0_, P_0_) to the intersection of D_2_ with S_1_ at point b (Q_1_, P_1_). The increase in price reflects both the quality premium and the effects of reduced quantity supplied, in spite of the premium. These changes imply a change in the total economic surplus in this market from area $\left\langle {{\text{faj}}} \right\rangle$ to area $\left\langle {{\text{dbj}}} \right\rangle$, comprising a change in consumer surplus (from area $\left\langle {{\text{faP}}_{{0}} } \right\rangle$ to area $\left\langle {{\text{dbP}}_{{1}} } \right\rangle$) and a change in producer surplus (from area $\left\langle {{\text{jaP}}_{{0}} } \right\rangle$ to area $\left\langle {{\text{gbP}}_{{1}} } \right\rangle$).^39^ Whether these changes amount to increases or decreases depends on the relative sizes of the shifts in supply and demand, and to some extent on the nature of the shifts (drawn here as divergent to some extent) and the slopes of the curves. If the upwards shift in the demand curve (reflecting the value of improved quality and quality assurance) is large relative to the upwards shift in supply (reflecting the costs of those improvements borne by producers), then both producers and consumers are likely to gain.

This analysis allows that yield restrictions are a costly way of accomplishing the desired quality improvement and quality signaling because, for the reasons discussed above (in the context of the discussion around Fig. 2), the single-valued maximum restriction will be sub-optimal for at least some producers in at least some years. The third supply function reflects the costs of supplying the same overall quality of wine, but doing so in the least-cost fashion by allowing each producer to optimize the quantity-quality trade-off, given the market premium for quality. We can imagine moving toward this outcome if the blanket yield restriction could be replaced with some more selective mechanism for signaling quality within the industry, or leaving it to the market given today’s comparatively effective market information mechanisms. If eliminating yield restrictions would allow an increase from S_1_ to S_2_ while preserving quality signaling, the new equilibrium would be at the intersection of D_2_ with S_2_ at point c (Q_2_, P_2_), yielding additional social benefits equal to area $\left\langle {{\text{gbch}}} \right\rangle$ in Fig. 3, definitely benefiting consumers (gains equal to area $\left\langle \text{P}_{1}\text{bcP}_{2} \right\rangle$) and potentially also benefiting producers.

So far, we have abstracted from the effect of policy-induced changes in the market for appellation wines on the market for table wines. To the extent that the restrictions are binding, and given barriers to increasing the total area of vineyard, yield restrictions do have the effect of reducing the volume of appellation wine produced. The consequences may be seen as a means of de facto price discrimination between consumers of appellation wine (of which less is produced) and table wine (of which potentially more is produced). In their analysis of the combined supply-restricting and demand-enhancing effects from quality regulations imposed by a PDO, Mérel and Sexton (2012, p. 570) find that “… the European GI regulations may in essence have replaced a pooling equilibrium with deficient product quality in the absence of intervention with a separating equilibrium involving a ‘generic’ product with fixed low quality and a GI product with excessive quality relative to the social optimum.” Mérel and Sexton (2012) did not model the efficiency losses from yield restrictions as quality controls, implicitly assuming that producers would supply the desired quality at minimum cost.

At some level, any technological regulation may be questionable as an economic policy tool; typically, economists will argue for the use of instruments that are closely targeted to the economic purpose and that allow market transactions to reveal opportunity costs (as in the use of cap-and-trade programs rather than technological regulations to manage emissions from oil refineries). This kind of thinking has been generally absent from discussions of EU wine policy. Our discussion of varietal and yield restrictions illustrates the general point in the context of the vineyard. Other examples in the context of the winery are also interesting (see, e.g., Appendix Box B-1).

### Enforcement Costs and Cheating

Our analysis thus far has abstracted from the issue of enforcement costs, the consequentially imperfect enforcement of regulations, and the lawbreaking that ensues. While we do not have any hard data on the extent of such behavior, we have evidence that some European wine producers do find it profitable to avoid complying with at least some of the regulations, some of which are not easy to enforce. It should be comparatively easy to enforce planting restrictions because area of vineyards is easy to observe. Nevertheless, as reported by Meloni et al. (2019, p. 637), the EU system of planting rights has seen considerable enforcement challenges: “In 2012 the European Commission imposed a fine of 250 million Euros on Greece, Italy, and Spain because of an estimated 120,000 hectares of vineyards planted illegally, that is, without planting rights; an area about the size of all vineyards in the Bordeaux region.”

Compared with area restrictions, especially for a perennial crop, yield and output restrictions are much more difficult to enforce, and more so in a case like this where the farmer produces a finished good, ready for the final consumer. Hard data on these phenomena are understandably elusive. However, producers have strong incentives to engage in black-market trade to meet shortfalls from allowable yield, or even exceed it. Indeed, based on informal advice from industry participants we guesstimate that, at least in some vintages, total production of wine in at least some PDOs may be as much as 30% greater than the official statistics based on permitted yields.

This is not a unique (or even unusual) situation, but very little of the published work in the economics of agricultural policy has paid serious attention to enforcement costs and cheating, and most of the published studies that do consider these aspects are conceptual rather than empirical. For example, Giannakas and Fulton (2000) analyze redistribution of economic surplus using agricultural output quotas in the presence of misrepresentation and cheating. As they show, when quotas are imperfectly enforced producers may find it profitable to produce more than their legally allowed amount. Compared with perfect enforcement, the greater quantity produced implies a smaller social cost of distortions associated with restricting output. Offsetting that saving, producers and sellers (and perhaps buyers) of undocumented production incur costs of bearing the risk of detection and punishment, and measures they take to avoid detection. In addition to these costs of compliance with the law (or cheating to evade it) we have costs of administration and enforcement of the policy.

These types of trade-offs are potentially important in the context of EU wine policy, but they are not represented in the analysis of Fig. 3, which is conventional in this respect. Once we have some kind of black market, the supply and demand curves drawn in Fig. 3 are no longer strictly relevant as such; nor is the concept of equilibrium with a single market clearing price. The quantity produced will fall somewhere between the quantity that would be produced with full enforcement of the policy and the quantity that would be produced with no policy restricting yields. As well as having implications for the net social costs of the policies, these economizing responses to the regulations will have complex consequences for the distribution of costs and benefits, all of which tends to weaken the case for using such policies.

### Governance and Regulatory Capture

As noted, along with changes in the definition of the spatial limits of the respective regions included in some appellations and other rules of the appellations, the number of appellations has been changing over time. Do we have too many appellations? Are the mechanisms for managing the structure of the appellations (including the spatial limits and other rules) open to capture by powerful vested interests? Do the decisions made by the producer organizations serve social purposes well?

The appellation system is administered by public authorities and producer organizations, and they are vulnerable to regulatory capture and government failure. This is a diverse industry that includes some very large producers, some of whom are politically and economically powerful, and may be active in multiple regions and wine GIs, along with a multitude of small producers, who might participate in policy processes only indirectly, through the cooperatives that process their wine. It is unlikely that any policy developed in the context of a producer organization will serve all of these diverse interests equally well, or that all of the interested parties will be equally effectively represented in the policymaking process.

The organizations (called Consorzi in Italy) governing wine GIs are democratic organizations, usually with three or four categories of members: grape growers, integrated grower-vintners, cooperatives, and commercial bottlers. Some decisions are determined by a vote of the industry members of the organizations; others by their elected representatives (board members). Although the voting power could be based on the volume of production—one ton (or one hl) = one vote—the votes are not distributed in this fashion in practice. Regulations exist to reduce the relative strength of the vote (per ton produced) of individual members who produce larger volumes and act independently, while cooperatives that represent many small producers are able effectively to exercise disproportionate influence on decisions.

These details may differ among Member States and organizations governing the GIs within them. Under Italian law, for example, whenever the board of a Consorzio proposes an action to reduce maximum yield or to introduce any other measure for reducing supply, the official procedure states that this action should be approved by the regional government and by a vote of an assembly of the members of the Consorzio. A proposal to reduce supply will inevitably have different—some favorable, some unfavorable—consequences for different industry participants. Hence, such proposals are always controversial, perhaps for good reasons.

Most individual wine GIs would not be seen as economically large producers able to exercise significant market power and profit substantially by restricting supply from the appellation. Even so, in recent times we have seen decisions taken within many wine GIs to introduce various measures to reduce the total production, without any pretense that the purpose was to enhance quality or collective reputation or overcome a lemons problem. Table 4 includes examples of policies introduced by the boards of the Consorzi of several Italian GIs under the system of authorizations for new plantings. In 2019, six of the nine Consorzi in Table 4 introduced a moratorium on new vineyards to be covered by the GI for the next 2 or 3 years. Others imposed tighter restrictions on the allowable grape-to-wine ratio for appellation wine which, combined with a restriction on grape yield and a fixed existing area of vineyard, acts effectively as a tighter restriction on volume of appellation wine to be produced.

**Table 4:** Actions taken in 2019 to protect Italian PDOs

Organization	Actions taken
*Consorzio di Tutela Prosecco DOC*	Reduction of the grape-to-wine ratio from 135 to 120 quintals
	Moratorium on new vineyards covered by the PDO until harvest of 2021
*Consorzio di Tutela Vini Valpolicella*	Reduction of the grape-to-wine ratio from 120 to 110 quintals
	Moratorium on new vineyards covered by the PDO until harvest of 2022
	Decrease from 65 to 40% in the proportion of wine grapes in Valpolicella DOC that may be used for Amarone and Recioto della Valpolicella
*Consorzio di Tutela Vini DOC Sicilia*	Reduction of the grape-to-wine ratio for Grillo grapes to 110 quintals
*Consorzio di Tutela Vini Orvieto*	Reduction of the grape-to-wine ratio from 80 to 75 quintals (since 2018)
	Moratorium on new vineyards covered by the PDO until harvest of 2021
*Consorzio DOC Delle Venezie*	Moratorium on new vineyards covered by the PDO until harvest of 2022
*Regione Emilia-Romagna*	Allowing 512,79 new hectares of vineyards for a total amount of 2,684 farms
*Consorzio Tutela Lugana*	Moratorium on new vineyards covered by the PDO until harvest of 2021
	Compulsory storage of the wine obtained from the 2019 harvest, until December 31, 2020 to support the market price
	Increased intensity of inspection by the consortium of vineyards until the 3rd year of age, to more closely monitor farmers’ operations and better enforce the consortium grape-to-wine ratio (70%)
*Consorzio di Tutela Barolo Barbaresco Alba Langhe e Dogliani*	Moratorium on new vineyards covered by the PDO until harvest of 2022
	Decrease in the grape-to-wine ratio from 80 to 70% for wines apt to be Barolo Riserva that benefits from MEGA (Additional Geographic Mention)
Consorzio Vini Alto Adige*	Reduction by 25% in the grape-to-wine ratio and thus in the total amount of production allowed
	Allowing a maximum of five winegrape varieties per suitable production area: a total of 86 terroir parcels waiting for MEGA recognition

Source: Created by the authors drawing on various online sources including: https://www.prosecco.it/wp-content/uploads/2019/08/Prime-Indicazioni_Tecniche_di-vendemmia-2019_v2.pdf, https://www.qualivita.it/wp-content/uploads/2019/07/20190731_RS_QN_VALPOLICELLA.pdf, https://www.agricultura.it/2019/07/26/doc-sicilia-consorzio-riduce-le-rese-delle-uve-grillo-a-110-quintali-ad-ettaro-per-la-vendemmia-2019/, https://www.ilmessaggero.it/umbria/orvieto_vino_crisi_la_regione_blocca_impianto_di_nuovi_vigneti_anni-4224332.html, https://dellevenezie.it/delibere-blocco-rivendica-per-il-triennio-2019-2022/, http://www.uiv.it/lemilia-romagna-ha-autorizzato-512-ettari-di-nuovo-vigneto/, https://winenews.it/it/il-lugana-ed-il-futuro-chiesto-lo-stoccaggio-del-10-della-vendemmia-2019-stop-a-nuovi-impianti_394501/, https://www.langhevini.it/barolo-nuovi-impianti-bloccati-per-tre-anni/

*Corriere Dell’Alto Adige; 7 settembre’19, pagina 11

In 2020, we have seen the implementation of even tighter yield restrictions on appellation wine as a response to the worsening market situation under the COVID-19 pandemic by some of the same Consorzi (e.g., Prosecco, Valpollicella, and Brunello di Mongtalcino). It cannot be claimed that the purpose of these yield restrictions was to enhance quality, and nor is it likely that they will accomplish that purpose. Rather, this can only be a crude mechanism for reducing the total quantity of quality wine in the face of an exacerbated supply–demand imbalance. Producers will seek and receive other emergency assistance, no doubt. Repeating the pattern of the past, European policymakers seem unlikely to take full advantage of the current crisis as an opportunity to embrace a shake-out of the industry and a shift towards a more sustainable long-run trajectory for the industry.

## Synthesis and Conclusion

European wine policy is centered on a system of GIs that entail significant technological regulations restricting the varieties that may be grown and the maximum yields per hectare, along with other rules regarding grape production and winemaking practice. In this article, we have raised questions about the extent to which these technological regulations are valuable today, given (1) the potential for alternative sources of information to solve the lemons problem, (2) evidence that the appellation system per se might not be effectively serving that purpose as well as it once did, and (3) evidence that some of the regulations impose significant social costs.

Since 1970, GIs for wine have operated within a broader European wine policy that sought to harmonize appellation rules and other policies across Member States and overcome some inherent structural problems. From the outset, EU wine policy was destined for trouble because the floor price for table wine was set above the market clearing price, causing excess supply, especially of the lowest-quality wine. This had long-lasting effects. The policy induced an increase in total productive capacity, especially in the production of lower quality, undifferentiated table wine, and the excess supply problem became worse over time reflecting longer-run supply response to an enduring change in incentives that contradicted market trends. The wine policy has evolved to address this structural issue, including very significant (and expensive) intervention to reduce excess supply, while seeking to preserve the essence of the appellation system. The GIs for wine have also evolved, but perhaps too slowly.

Today, limitations on the varieties that may be grown within a particular location to qualify for premium status seem excessive in a world where varietal labels can be used to signal that aspect of the production process, and where the economic environment of production is changing in ways that exacerbate the costs of inflexible restrictions on varietal choice. We have already seen some initiatives in relation to varietal innovations—as a response to climate change—that would have been unthinkable a decade or so in the past. However, we also see strong headwinds of opposition to the most promising methods for making varietal innovations to address emerging environmental, marketing, and policy challenges—such as GMOs, gene-edited plants, or hybrids.

Several questions arise in the context of yield restrictions. First, do they effectively result in an increase in average quality that enhances the collective reputation of wine from a PDO or PGI? Second, what are the implications of heterogeneity of quality, inherent in the design of the policy that appears to ignore this aspect? Even if yield restrictions are effective at enhancing average quality, in a highly heterogeneous and variable production environment a blanket yield restriction is an economically ill-suited instrument for enhancing and signaling quality. Third, is the true purpose of yield restrictions supply control? If so, this should be made explicit and evaluated, since yield restrictions are likely to be an economically inefficient instrument for that purpose, even if combined with planting restrictions.

The fact that the restrictions on yields and other rules are imperfectly enforced is a mixed blessing. It reduces the harm that is done when rules are inappropriate and costly. However, at the same time it undermines the purpose of policies that are meant to serve a broader social purpose, and at some level it undermines the social fabric more broadly, by creating a culture of lawlessness and a loss of trust in institutions. Some industry participants may be dissatisfied with the system for other reasons also related to lack of confidence in the institutions and fairness.

Governance and regulatory capture is a neglected topic that warrants further study. The governance structures for the wine GIs might be contributing to forms of government failure in that the organizations might be devising and implementing policy in ways that do not represent the interests of producers adequately or serve the broader society well. A related issue is that the governance structure of the GIs might be contributing to the inertia and slowness to reform some rules and regulations that no longer serve the broader society well, though they may still serve some narrower vested interests.

In this article we have conjectured that some aspects of the wine appellation system that were once economically useful might no longer pass the cost–benefit test in today’s much-changed wine world. Our analysis shows that the technological regulations restricting yields and varieties are of dubious value in today’s circumstances. As well as saving the costs of resource distortions, Europe could save the heavy burden of regulatory enforcement and compliance. In the other wine-producing countries that are competing well against the EU, GI systems for wine operate successfully without restricting varietal choices for producers and without imposing yield restrictions—used in European GIs ostensibly to enhance quality but more clearly as a clumsy and economically inefficient way to restrict supply. Clearly, it would be economically better for the EU to abandon yield restrictions as a way of trying to manage quality and quantity, and to find ways to sustainably support European wine producers that do not encourage them to produce wine that the market manifestly does not want. Wine policies introduced in the 2009 CAP reform, and subsequently, seek to facilitate structural change and enhance demand. Policies of this type could continue to complement a better-conceived system of support for the industry and quality signaling for consumers.

## Appendix Box B-1 Arbitrary Regulations on Use of Water and Sugar in Winemaking

Imagine two wine economists, one from the Old World (say, northern France—call him Pierre) and the other from the New World (say, California or Australia—call him Jim). In Pierre’s world it is sacrilegious to add water to wine in the wine-making process; the EU rules of wine-making absolutely forbid it—in Europe, nowadays, the only way water can be made into wine is by the grapevine! Conversely, in Jim’s world it is permissible to add water if necessary to complete the fermentation process; sometimes there is simply too much sugar and the alcohol kills the yeast. In Pierre’s world, the opposite problem arises. The grapevine does not produce enough sugar to meet the legal minimum (and desired) alcohol content, and it is common in Europe to add considerable amounts of sucrose (from sugar cane or sugar beet) to strengthen the wine. In contrast, this practice—chaptalization—is prohibited (heresy!) in California, though not in some cooler U.S. states, such as Oregon. California winemakers may add grape juice concentrate, as may winemakers in Italy—who used significant quantities of subsidized grape must at times (which could have come from outside the DOC or DOCG)—but not those in France.

A reasonable person might conclude that specific technological regulations like these are not based in any natural law related to wine per se, but rather are a little capricious and somewhat arbitrary—more like a matter of convenience, accommodating the applicable environmental conditions that vary from place to place—which might account for the highly moralistic tone assumed in discussion of the sanctity of such rules. California has abundant sunshine and heat, and consequently permits the addition of water, to reduce excessive sugar concentration, but does not permit the addition of sugar. France has the converse situation, especially in the cooler northern parts, in that grapevines do not produce enough sugar. In making fine French wine it is unthinkable to dilute grape juice with water, and it is de rigueur to strengthen it with sugar—so long as the sugar is derived from an entirely different species! We can wonder if climate change might bring about some flexibility in these fundamentally climate-driven rules.

## Appendix Box B-2 Information Asymmetries and Signaling Issues in Wine Markets

It is commonly claimed that wine is an experience good, implying that although consumers cannot judge wine until they taste it, they can judge wine when they do taste it, and it is these sensory attributes of wine that really matter for their appreciation and valuation of the product. Much evidence exists to belie these seemingly straightforward ideas. The *Journal of Wine Economics* has published numerous articles demonstrating the fallibility of wine judges and other experts (see, e.g., Hodgson 2008, 2009). If the acknowledged experts cannot consistently assess wine, why should an amateur be any more successful? Other articles in the same journal show that wine is a horizontally differentiated good in that different consumers like and value different attributes (e.g., Goldstein et al. 2008), and that sensory perceptions and willingness to pay for wine are significantly affected by context including the price of the wine, scores from expert raters, information on the label about the wine, and other environmental factors (e.g., Goldstein 2019). The market for wine consequently abounds with information asymmetries and complex signaling problems.

Wine producers understand these attributes of their customers and the marketplace in which they operate, and they produce and market their wines accordingly. Some strange outcomes emerge. For example, because consumers have come to believe that wine with screw-caps is inferior, producers of better wines are likely to use corks to signal high quality, even if the cork itself is an inferior closure and more likely to result in damaged wine. Likewise, if consumers believe that particular aspects of the production process (e.g., old vines, low-yielding, biodynamic) result in higher quality wines (whether because it is inherent in the wine, or because of some implied scarcity or exclusiveness), producers may accordingly find it profitable to restrict yields so they can use low-yield as a signal of quality, even if restricting yields does not actually enhance quality. Some aspects of the PDO rules seem to be contributing to these outcomes—not just in Europe—and the rules might be perpetuated for the same kind of circular cause-and-effect reasons. Indeed, some PDOs require corks to be used.

## Footnotes

1 GIs and the associated protected naming rights apply to many other food and non-food products around the world. Applications of GIs to wine in Europe date back to the seventeenth and eighteenth centuries and wine is still the predominant user of GIs globally (Meloni et al. 2019). GIs were established for products like ham and cheese in the EU in 1992 (EU Reg. 2081/1992) and, since 2008, the system of GIs for wine in Europe now fits within the broader EU-wide system of GIs under the single Common Market Organization (EU Reg. 479/2008 Art. 34).

2 This analysis abstracts completely from many other aspects of government policy pertaining specifically to wine production, consumption and trade, such as regulations and taxes on domestic sales whether applied for public health or other reasons; tariffs and other trade policies, some of which relate to GIs and other intellectual property; antitrust laws and other commercial policies.

3 Munsie (2002), Haeck et al. (2019) and Mérel et al. (2019) provide details on the AOC system and its history. The first French law on viticultural designations of origin dates to 1905 (Chevet et al. 2018, pp. 69–73).

4 The systems of GIs chosen by Italy, Spain, and Portugal were modeled on that of France. Germany opted to use a different system altogether, and Austria followed (see Munsie 2002 for details). Table wine is ruled out in Germany since it is not legal to produce wine outside the regions associated with its 13 PDOs and 26 PGIs (German Wine Institute 2020).

5 Gaeta and Corsinovi (2014) describe both an old quality pyramid (p. 187), reflecting the 1999 Regulations (which amended the pyramid established in 1970), and a new quality pyramid (p. 191), reflecting the 2008 Regulations and subsequent amendments.

6 The EU imposes a minimal set of regulations on production for any wine. These regulations apply to wines without a GI. In contrast, while the EU sets the minimum legal framework, it is left up to each Member State to determine the details of its system of classification and control for appellation wines within that framework.

7 Some further differentiation is found within PDOs. In Italy, for example, within DOC (corresponding to PDO) wines, there is another category represented by DOCG wines (Controlled and Guaranteed Designation of Origin); and within DOC and DOCG wines, sub-appellations and sub-areas are defined (such as Valpolicella Classica within Valpolicella).

8 The CMO for wine was a compromise between the positions of Italy and France. Indeed, as described by Munsie (2002, p. 11) “… the main conflict that had to be resolved in bringing together these countries [to create the EEC] was the integration of the French and Italian wine industries. Although France and Italy were the two biggest wine producing countries, the ways in which they were organized were polar opposites.” The French feared that Italian wine would flood the market and cause prices to plummet. These fears were to be realized within a few years under the CMO.

9 “Regulation 816/70 contained the basic provisions to implement the common organization of the wine market, while Regulation 817/70 contained provisions specifically aimed at quality wines” (Munsie 2002, p. 12). These two parts of the wine industry were regulated separately, in this way, until the 1999 reform.

10 Our description of the historical development of European wine policy draws on Munsie (2002), Meloni et al. (2019), and Pomarici and Sardone (2020), in particular, but also Gaeta and Corsinovi (2014) and Meloni and Swinnen (2016), among others. The European Commission (2004) provides a detailed ex post evaluation of the wine CMO through 2002. More recent coverage is provided by Agrosynergie (EEG) (2019).

11 Further changes are envisioned in the 2018 proposal for CAP post-2020 (see Pomarici and Sardone 2020), but these ideas might have been overtaken by subsequent events including Brexit, the Trump tariffs, and the COVID-19 pandemic.

12 As described by Meloni et al. (2019), since the 1990s pressure to reform the EU wine policy grew from a loss of competitiveness, high budget costs, and new challenges such as climate change and demands for sustainable farming. However, substantive policy reform has been resisted stiffly by producers, as exemplified by the failed attempt at radical reform in 1994 (see also Pomarici and Sardone 2020).

13 Much European wine is fortified by the addition of sucrose (from sugar beet or sugar cane) or rectified concentrated grape must (a sugary solution derived from grape juice)—as required to achieve minimum alcohol concentration required by EU and appellation rules, as well as for commercial reasons. The addition of sucrose is forbidden in Italy, Spain, Portugal, Greece, Cyprus, or Southern France. Regulations have changed over time regarding the use of concentrated must—currently allowed only in Italy, Spain, Portugal and Greece. In Italy, subsidies on use of grape must for fortifying wines were helpful both in reducing the problem of excess supply and in assisting financially straitened producers in the south, while generally supporting producers.

14 On October 2, 2019, the World Trade Organization (WTO) authorized the United States to impose duties on $7.5 billion worth of European goods after the EU was found guilty of unfair subsidies associated with Airbus. On the following day, the Trump administration announced 25 percent tariffs on a wide range of European products, including wine with less than 14% alcohol by volume, which went into effect October 18, 2019.

15 Responding to this crisis, on May 4, 2020, the European Commission published several measures, based on available support under the CAP and the CMO, to help rebalance supply of wine to adjust to the shifting demand caused by the COVID-19 crisis. These measures include private storage aid and flexibility in the implementation of market support programs—including a provision for Member States to include by-product distillation for industrial use under their National Support Programs (NSPs). On July 7, 2020, the European Commission announced additional exceptional measures, including increases in the EU’s contributions for NSPs (up to 70%) and advanced payments for crisis distillation and storage, and called for proposals for promotion programs.

16 EU rules on planting rights are based on French regulations tracing back several centuries, with royal edicts restricting the planting of vines in the Bordeaux region as early as 1725. After a liberal period following the French Revolution, restrictions were again imposed in the 1930s and further strengthened in the postwar period. These regulations served as the basis for regulations in the EU, initially introduced in 1970 and further strengthened in 1976 and 1984 (see Deconinck and Swinnen 2015; Meloni et al. 2019).

17 An increase of 0.9 HL/ha/year over seven years, 1977–84 amounts to 6.3 HL/ha on a base of about 35 HL/ha/year.

18 AOC wine producers wanted to market their wines as distinctly different from table wine; they were not so much interested in reduced volume from those areas, and not so much changing practices as codifying them. At the same time, producers of table wine sought and were granted a minimum (floor) price, which was conditional on some measure of supply control. See Loubère (1990).

19 The GIs for wine can be viewed as an element of the broader EU system of GIs for agri-food products (see, e.g., Moschini et al. 2008) for which, in its effects, the whole may be greater than the sum of its parts—if, for example, the use of GIs to differentiate and signal quality attributes for EU wine has spillover benefits across other EU agri-food products, and vice versa. Here we ignore those potential cross-commodity spillovers and focus on the direct consequences of the rules and regulations within the GIs for wine in Europe.

20 Menapace and Moschini (2012) distinguish between GIs, which create collective reputation by signaling intrinsic quality associated with geography or terroir, and trademarks, which are firm-specific and private and can be used in a complementary fashion with GIs.

21 Hedonic studies by economists have produced a mixture of results on the value of terroir (see, e.g., the extensive listing of studies and discussion by Haeck et al., 2019). Nevertheless, there appears to be a clear general association of quality and price with the place of production for wine, and producers perceive returns to creating a collective reputation associated with a PDO. See Frick and Simmoins (2013) and studies they cite regarding the economics of collective reputation for wine.

22 International trade issues have arisen over the use of names such as Champagne or Chianti to describe wines produced in other regions, in particular by New World producers. These issues have been addressed in various international fora, such as the WTO and the OIV, and are the subject of various bilateral and multilateral agreements. The relevant EU regulations were revised in 2008 and 2013 (Reg. 4798/2008 and Reg. 1308/2013).

23 For example, the Bordeaux region produces both Château Petrus (in Pomerol) and Château Cheval Blanc (in Saint-Émilion). It does not seem likely that either of these iconic wine labels benefits much, nowadays, from being associated with the region in which it is produced; though they might have benefited in the past, and they might confer a halo effect on their less-highly rated neighbors.

24 AVAs are defined geographic areas that may be quite large and cross state or county lines, or may be quite small and lie within a county or, in some cases, another AVA. The Napa Valley AVA is, for instance, a large AVA located within Napa County. The Oakville AVA is a much smaller AVA that is located within the Napa Valley AVA. In contrast, the Carneros AVA is a defined AVA in the southern portion of Napa and Sonoma Counties.

25 Wineries may identify the grapes used in a wine as coming from an AVA if 85 percent of the grapes were grown in the AVA and the wine is fully finished in the state where the AVA is located.

26 Between 1970 and 2019, California’s total area of grapes used for wine making at least doubled (USDA/NASS 1970–2019). Meanwhile, the industry shifted to premium varieties and toward premium locations in the cooler coastal valleys. This shift to quality wine was probably larger than the shift to quality wine in France, and it was achieved by the unfettered working of the market mechanism, without central direction or massive government expenditure.

27 Using data on 8800 California wines, Kwon et al. (2008) found evidence that narrower GIs were correlated with an increase in perceived quality, as indicated by the price of wine.

28 As a component of this problem, there is not a clear and unambiguous ranking of quality—as perceived by consumers and reflected in prices—as we go up the quality pyramid (see, e.g., Appendix Box B-2).

29 The first Appellation (zone) in Champagne was delimited in 1908. In 1911, under pressure from the excluded areas, the government created two zones: the original (1908) Champagne Appellation was called “Champagne AO Zone 1” and a second Appellation was called “Champagne AO Zone 2.” As of 1927, Champagne Zone 1 and Champagne Zone 2 were regrouped into a single Appellation (see Haeck et al. 2019).

30 Until recently, Prosecco was protected as an appellation for DOC wine produced in a relatively small geographic area within the Veneto region of Italy. In response to a perceived market opportunity, some producers sought PDO status for Prosecco under European law, and they succeeded. The upshot was three major changes, implemented in the 2009 vintage. First, a new DOC for Prosecco was established over a vastly expanded region extending almost to Trieste, so as to be able include the town of Prosecco—as required under European law if Prosecco is to be used as the place-name for the GI. Second, the variety Prosecco was renamed Glera, still the primary variety that can be used to make Prosecco wine, such that the word Prosecco now serves exclusively as a GI. Third, the original areas were promoted to DOCG status—advantaged in principle, but with their collective brand to be somewhat undermined by the much lower-cost DOC Prosecco produced from grapes grown on the plains with much higher yields and using mechanized production systems.

31 Distretto Del Conegliano Valdobbiadene (2018) documents an increase in exports of Prosecco to the UK market from 482,288 bottles in 2003 to 7,444,945 bottles in 2017, increasing from 4.5 to 20.5% of total UK imports of PDO sparkling wine at the expense of Cava and Champagne.

32 As described by Meloni and Swinnen (2013), between 1931 and 1935, a series of laws (Statut Viticole) aimed at controlling wine supply were introduced, such as an obligation to store part of the excess production (blocage), obligatory distillation of surpluses, a ban on planting new vines, and premiums for grubbing up of overproductive vines. The introduction of the AOC system helped, but these other policies were more immediately important for reducing the wine glut.

33 For example, the new wine, Terregiunte (connected-earths), born from the collaboration of two well-known corporate brands, Masi from Veneto and Vespa from Puglia, combines wine from two separate PDOs and therefore is entitled to use only the generic name Italian wine, even though it is premium wine and priced accordingly.

34 Crosses between European *Vitis vinifera* and American varieties (inter-specific hybrids) became popular in Europe initially as a response to phylloxera. As discussed by Meloni and Swinnen (2013), these hybrids were a target for policy as a contributor to overproduction of lower-quality wine. Even though they were highly regulated under the AOC system, by the end of the 1950s hybrids still made up one-third of France’s vineyard area (and 42% of table wine production); however, with the subsidized replacement of some 325,000 ha of hybrids during the 1970s and 1980s, by the end of the 1980s this share had been reduced to less than 3% (Meloni and Swinnen 2013). It subsequently shrank to less than 1%.

35 Genetically engineered (GE) crops often have a smaller environmental footprint than their conventional counterparts (see, e.g., Wu 2006; Klümper and Qaim 2014). Conventional cross-breeding and selection involves multiple uncontrolled changes, *mutatis mutandis*, whereas gene-editing and transgenic insertion involve single controlled changes, *ceteris paribus*, and is less risky in that sense (see, e.g., Qaim 2009).

36 Lower yields might reflect smaller berries, and a higher ratio of skin (which provides flavors) to pulp, and consequently more-intensely flavored wines; they might reflect delayed harvest, which can result in riper and more concentrated fruit flavors as grapes become desiccated; they might reflect pruning, green thinning, or other management practices that reduce the total crop but increase homogeneity at harvest.

37 In this depiction, we assume producers are well informed and rational, so they achieve outcomes on the frontier, which represents the maximum attainable quality for given yield, and vice versa. As noted above, the introduction of technological regulations under appellations may have had significant didactive effects, which could be depicted as moving producers to the frontier from technically inefficient outcomes below it.

38 As discussed in the context of Fig. 2, if producers can influence quality by varying factor proportions, we would expect the introduction of a restriction on yield to induce an increase in average quality.

39 The demand reflects all consumers, whether from within the jurisdiction imposing the regulations (e.g., a member of the EU) or elsewhere (e.g., other countries). For some purposes, it might be appropriate to conduct a welfare analysis partitioning the demand (and consumer surplus) between domestic and foreign consumers. This may change the calculus in favor of the policy since restricting supply is a mechanism for exercising market power in trade and extracting economic surplus from foreign consumers, even if also at the expense of domestic consumers—see, e.g., Johnson (1965) and Sumner and Alston (1984) for an application to U.S. tobacco.
